# Development and Application of an In Vitro Pollen Viability Assay for Comparative Safety Assessment of Transgenic Alfalfa (*Medicago sativa* L.)

**DOI:** 10.3390/plants14193070

**Published:** 2025-10-04

**Authors:** Yuxiao Chen, Xiaochun Zhang, Jiangtao Yang, Diandian Guo, Xujing Wang, Zhixing Wang

**Affiliations:** Biotechnology Research Institute, Chinese Academy of Agricultural Sciences, Beijing 100080, China; 821012410033@caas.cn (Y.C.); 82101221024@caas.cn (X.Z.); jt_y1990@163.com (J.Y.); diandiang1205@163.com (D.G.)

**Keywords:** pollen viability, biosafety, alfalfa, protocol

## Abstract

Alfalfa (*Medicago sativa* L.) is a vital global forage crop. Transgenic technology promises enhanced yield and quality, but requires rigorous environmental risk assessment, particularly regarding pollen-mediated gene flow, for which standardized protocols are lacking. Based on an optimized in vitro culture medium, this study developed a method to assess alfalfa pollen viability. Using a single-factor experimental design, key assessment parameters were established at 1/4/8 h and 20/30/40 °C. A comparative analysis revealed no significant difference (*p* > 0.05) in pollen viability between the transgenic line SA6-8 and its non-transgenic parent “ZM-1” within this evaluation system. This result indicates that the genetic modification did not impact the pollen viability of SA6-8. By establishing this in vitro germination-based pollen viability assessment system and comparatively analyzing pollen viability between transgenic alfalfa and its non-transgenic parent under diverse environmental conditions, our approach provides crucial insights for optimizing transgenic alfalfa planting strategies and strengthening biosafety review protocols.

## 1. Introduction

Since the initial commercialization of genetically modified (GM) crops in the 1990s, biotechnology has emerged as a central driving force in global agriculture, addressing pressing challenges of food security [1]. By enabling the precise introduction of foreign genes, GM technology has achieved remarkable advances in insect resistance, herbicide tolerance, abiotic stress resilience, and nutritional enhancement—achievements often beyond the reach of conventional breeding [2,3,4,5]. These innovations have not only significantly increased yield per unit area and improved crop quality but have also played a pivotal role in reducing pesticide use, conserving arable land, and lowering agricultural carbon emissions, offering a powerful solution for sustainable agriculture amidst global population growth [6].

Alfalfa, renowned as the “King of Forages,” is one of the world’s most important perennial leguminous crops [7]. Cultivated on over 30 million hectares globally, it serves as a cornerstone of sustainable livestock production due to its high protein content (18–22%), exceptional biomass yield, and valuable ecosystem services such as nitrogen fixation [8]. However, alfalfa production faces increasing challenges: abiotic stresses and biotic stresses consistently constrain its yield and quality; conventional breeding is slow and inefficient, struggling to meet rapidly growing demands; additionally, high lignin content reduces forage digestibility, while over-reliance on herbicides imposes economic and environmental costs [9,10]. Biotechnology, particularly genetic engineering, offers powerful tools to address these bottlenecks. Researchers have successfully developed numerous transgenic alfalfa lines with superior agronomic traits and elucidated multiple stress resistance regulatory networks in alfalfa through biotechnology approaches, thereby providing enhanced options for molecular breeding [11,12,13,14]. For instance, alfalfa germplasm with traits such as high yield, insect resistance, herbicide tolerance, and salt and drought tolerance [15,16,17,18]. These breakthroughs indicate tremendous application potential for transgenic alfalfa.

However, the application of GM technology is not without controversy [19]. Its potential risks necessitate that any transgenic crop undergo rigorous, multidimensional safety assessment prior to environmental release [20]. This evaluation system serves as a critical bridge between laboratory research and large-scale application, aiming to systematically assess the potential impacts of genetically modified organisms (GMOs) on human health, the environment, and biodiversity. It ensures a balance between technological innovation and biosafety, safeguarding long-term stability in agricultural production and ecosystems, and is fundamental to gaining public acceptance and international market access [21].

Biosafety assessment of GMOs typically comprises two main pillars: food safety and environmental safety [22,23]. Environmental risk assessment focuses on the behavior and impact of GM crops within natural ecosystems, among which pollen-mediated gene flow is one of the most concerning processes [24]. This refers to the transfer of transgenes from GM crops into wild relatives or conventional varieties via pollen dispersal [24]. This process may lead to a range of ecological consequences, such as the emergence of more adaptable “superweeds,” erosion of genetic integrity and diversity in wild populations, adverse effects on non-target insects and biological communities, as well as intellectual property disputes and international trade conflicts [25,26]. Therefore, accurately assessing the potential for gene flow is crucial for environmental risk classification and the development of cultivation strategies for GM crops.

The probability and extent of gene flow fundamentally depend on pollen viability, dispersal distance, and adaptability under various environmental conditions [27]. Among these, pollen viability is the prerequisite for successful pollination and hybridization. Current methods for assessing pollen viability primarily include staining assays and in vitro germination. However, staining methods only indirectly infer viability by detecting enzyme activity or membrane integrity, are prone to false positives/negatives, and fail to reflect the actual germination capacity on stigmas [28]. In contrast, in vitro germination, which simulates the stigmatic environment by observing and quantifying pollen tube growth on artificial culture media, is widely recognized as a more direct, reliable, and functionally relevant “gold standard” method for evaluating pollen competitive fitness [29].

Nevertheless, alfalfa is a typical outcrossing species with open flowers, high pollen production, and extensive open-field cultivation. Its pollen can be transported over long distances by wind and insects. Therefore, conducting stringent environmental risk assessments is imperative. Unfortunately, although pollen viability assessment is central to risk evaluation, a standardized protocol for transgenic alfalfa is currently lacking internationally. Specifically, a standardized in vitro germination method capable of simulating complex field conditions (e.g., temperature fluctuations, post-anthesis time) remains unavailable. Thus, this study aims to address this critical gap.

Our objectives were threefold: first, to establish a highly efficient and robust in vitro pollen germination system for alfalfa through single-factor optimization; second, to systematically investigate the effects of key environmental factors (temperature and post-anthesis time) on pollen viability to determine optimal assessment conditions; and third, to apply this established protocol to compare pollen viability between transgenic alfalfa SA6-8 and its non-transgenic recipient. This study provides essential technical criteria and data for the scientific assessment of gene flow potential in transgenic alfalfa, ultimately supporting scientific decision-making for its environmental risk assessment and commercial application.

## 2. Results

### 2.1. Microscopic Observation of Pollen Morphology

**Figure 1:**
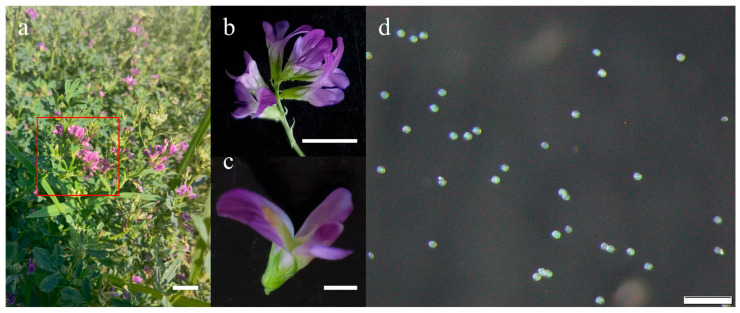
(**a**) Pollen sampling and initial characterization in alfalfa plants under field conditions. The red box indicates the sampling region, all the pollen used in the experiments was collected from upper flower buds nearing anthesis. Scale bar: 1 cm. (**b**) A representative flower used for pollen collection. Scale bar: 1 cm. (**c**) Anthers releasing pollen immediately after gentle pressure were applied to the keel petal. Scale bar: 2 mm. (**d**) Alfalfa pollen grains suspended in distilled water. Scale bar: 200 µm.

**Figure 2:**
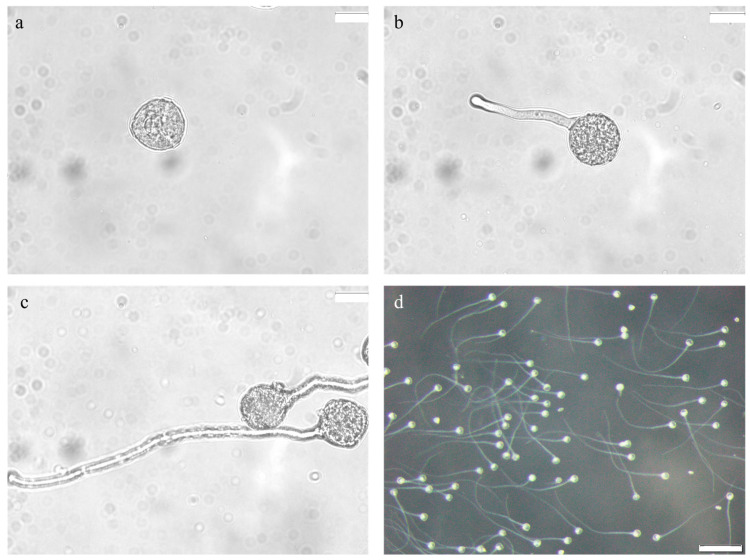
Microscopic observation of alfalfa pollen germination in vitro: (**a**) Alfalfa Pollen Morphology. Scale bar = 20 μm. (**b**) Alfalfa pollen after 15 min of in vitro culture in germination medium, Scale bar = 20 μm. (**c**) Alfalfa pollen after 1 h of in vitro culture in germination medium. Scale bar = 20 μm. (**d**) Alfalfa pollen after 1 h of in vitro culture in germination medium. Scale bar = 200 μm.

The screened anthers with uniform initial status were used for the subsequent experiments. Fresh pollen grains were immediately immersed in distilled water for morphological observation under a microscope. The pollen exhibited a nearly spherical shape, often appearing trilobate to sub-spherical from certain angles (Figure 1d). The structural integrity and morphological uniformity confirmed the suitability of the pollen samples for subsequent experimental stages. Germination assays conducted in medium (100 g/L sucrose, 200 mg/L boric acid, 60 mg/L calcium chloride, 10 mg/L monopotassium phosphate, 10 mg/L magnesium sulfate) showed that at 30 °C, alfalfa pollen tubes grew to twice the pollen diameter (62.52 μm) within 10–15 min and extended to approximately seven times the original diameter (221.33 μm) after 1 h (Figure 2a–c). These developments were clearly observable under microscopy. Therefore, a 1 h incubation period in the germination medium was adopted for all subsequent observations and imaging.

### 2.2. Screening of the Initial Medium

Through a systematic review and optimization of existing pollen culture media, we first identified sucrose (as carbon source), boric acid (H_3_BO_3_), and calcium chloride (CaCl_2_) as essential components of the in vitro germination medium for alfalfa pollen [30,31,32]. Both boron and calcium play critical roles in pollen germination: boron significantly promotes pollen tube growth by forming borate ester complexes with pectin components in the pollen cell wall, thereby enhancing wall elasticity and plasticity; calcium ions act as an important second messenger during pollen–stigma interactions by responding to calcium signals released from the stigma, establishing a concentration gradient that guides directional pollen tube elongation through chemotaxis [33,34,35]. Based on the identification of these essential components, we further employed an orthogonal experimental design to optimize their concentrations. This systematic approach evaluated the effects of different concentrations of sucrose (50, 75, 100, 125, 150, 200, and 400 g/L), boric acid (100, 200, and 300 mg/L), and calcium chloride (30, 60, and 90 mg/L) on the in vitro germination rate of alfalfa pollen. The results showed that the highest pollen germination rate was achieved at a sucrose concentration of 100 g/L (Medium M3), indicating that this concentration provides the optimal carbon source supply for in vitro alfalfa pollen germination (Table 1). Further optimization of the trace element ratios demonstrated that a combination of 200 mg/L boric acid and 60 mg/L calcium chloride (Medium M12) yielded the highest germination rate of 93.48 ± 1.62%, reflecting the most effective synergistic promotion effect (Table 2). This systematic optimization strategy ensures reproducible and quantitative analysis of alfalfa pollen viability under controlled conditions.

### 2.3. Further Optimization of Pollen Germination Medium

To enhance the optimization of the culture medium, a methodical evaluation of supplementary micronutrients was carried out. Monopotassium phosphate (KH_2_PO_4_) contributes critical phosphate groups for ATP production and potassium ions (K^+^) to modulate osmotic regulation, whereas magnesium sulfate (MgSO_4_) supplies Mg^2+^, serving as a cofactor for energy-metabolizing enzymes and reinforcing cell wall architecture via pectin cross-linkage [36,37,38]. The coordinated effects of these compounds facilitate efficient pollen tube extension by supporting redox balance and cytoskeletal organization. The application of KH_2_PO_4_ and MgSO_4_ at various concentrations (0, 10, and 20 mg/L) resulted in an increased germination rate of isolated alfalfa pollen (Table 3). The most favorable results for both compounds were observed at 10 mg/L. Accordingly, an optimized medium formulation—consisting of 100 g/L sucrose, 200 mg/L H_3_BO_3_, 60 mg/L CaCl_2_, 10 mg/L KH_2_PO_4_, and 10 mg/L MgSO_4_—was established and employed in all following experimental procedures (Figure 2d).

### 2.4. Effect of Temperature Treatment on Pollen Viability

**Figure 3:**
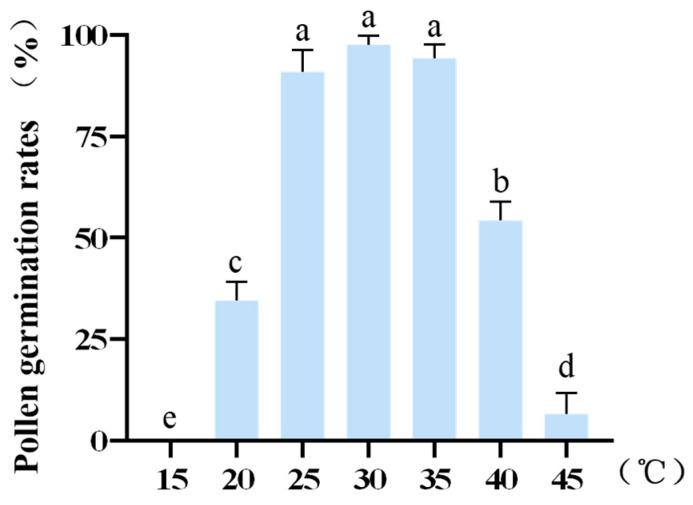
Pollen germination rate in response to different temperature. Different letters indicate statistically significant differences (*p* < 0.05), and error bars represent standard deviations.

In natural environments, pollen germination often occurs under fluctuating temperatures, and temperature is a critical factor significantly influencing pollen survival time in vitro [39]. To simulate the temperature variations occurring during anther dehiscence and pollen dispersal under natural conditions, this study systematically analyzed the in vitro germination rate of alfalfa pollen under different temperature treatments. Pollen samples were incubated at various temperatures (ranging from 15 °C to 45 °C) in precision-controlled incubators, and germination rates were quantified via microscopic examination (Fig. A2). The results revealed a typical triphasic temperature response pattern: germination was completely inhibited at 15 °C; within the 15–30 °C range, the germination rate increased with temperature and peaked at 30 °C; while a significant negative correlation was observed between 30 °C and 45 °C (Table A4). Accordingly, 30 °C was identified as the optimal germination temperature and used as the control condition in subsequent experiments. Further analysis indicated a relatively low germination rate at 20 °C. No significant differences (*p* > 0.05) in pollen viability were observed at 25 °C and 35 °C compared to 30 °C, whereas statistically significant decreases (*p* < 0.05) were detected at 15 °C, 20 °C, 40 °C, and 45 °C (Figure 3). This suggests that as an outcrossing species, alfalfa pollen exhibits broad temperature tolerance and high germination stability within the 25–35 °C range. According to meteorological records from the experimental site between 2022 and 2024, the flowering period of Medicago sativa occurs from May to October, during which temperatures range from 14 °C to 41 °C, with average temperatures between 20 °C and 36 °C. Based on both experimental results and field climate data, three temperature points—20 °C, 30 °C, and 40 °C—were selected to represent low-temperature stress, optimal condition, and high-temperature stress, respectively. This three-tier temperature evaluation system was established to systematically assess the environmental resilience of alfalfa pollen under controlled in vitro conditions.

### 2.5. Effect of In Vitro Duration on Pollen Viability

**Figure 4:**
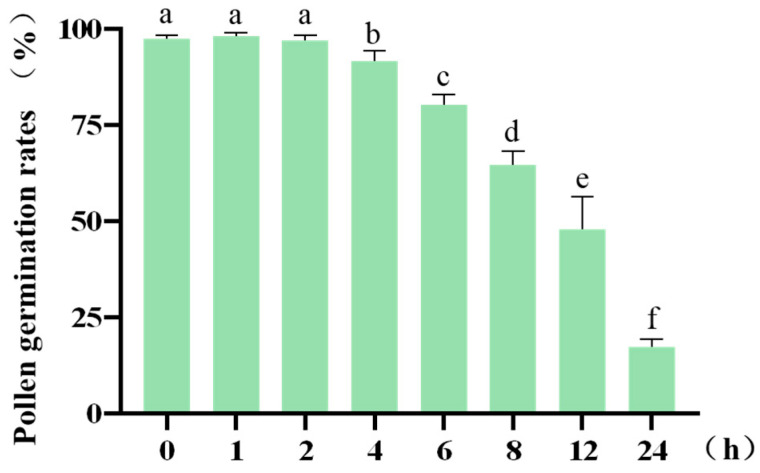
Pollen germination rates following varying post-excision durations. Different letters indicate statistically significant differences (*p* < 0.05), and error bars represent standard deviation.

Pollen-mediated gene flow, facilitated by wind or insect dispersal, enables cross-pollination. The effective distance of transgene escape is critically dependent on pollen viability under in vitro conditions [29]. To evaluate the effect of post-excision time on pollen viability, we systematically analyzed germination rates at multiple time points after isolation (0, 1, 2, 4, 6, 8, 12, and 24 h). The results indicated a time-dependent decline in pollen germination efficiency (Table A5). No significant differences (*p* > 0.05) in germination rate or viability were observed within the first 2 h compared to freshly collected pollen (0 h). However, a statistically significant reduction (*p* < 0.05) in germination rate was detected starting at 2 h, followed by a continued decrease over time. A sharp decline in viability occurred between 6 and 12 h after excision (Figure 4). Based on these findings, and considering practical applicability, three critical time points—1, 4, and 8 h post-excision—were selected for subsequent assessment of pollen environmental stress tolerance.

### 2.6. The Transgenic Event in SA6-8 Did Not Affect Pollen Viability Relative to ZM-1 Controls

To systematically evaluate the potential impact of biotechnology-mediated genetic modification on pollen viability, a comparative study was conducted using transgenic alfalfa (SA6-8) and its non-transgenic parental line (ZM-1) under strictly controlled environmental conditions. Pollen grains from both genotypes were collected at the same developmental stage and subjected to in vitro storage for 1, 4, and 8 h, simulating the possible time interval between anther dehiscence and pollination under natural conditions. Subsequently, the pollen was incubated at 20 °C, 30 °C, and 40 °C for 1 h to mimic low, optimal, and high temperature regimes that may occur in field environments.

**Figure 5:**
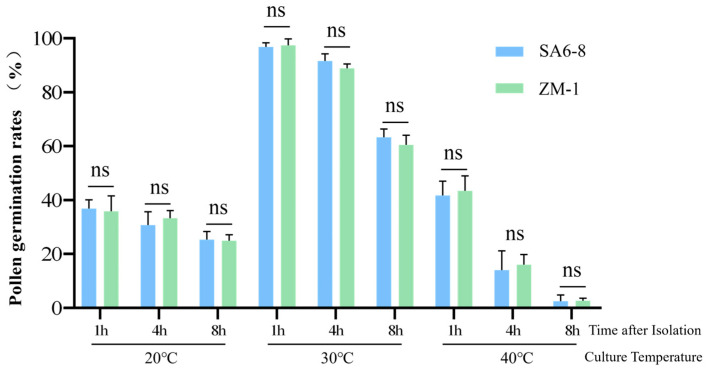
Pollen viability of transgenic alfalfa SA6-8 and non-transgenic ZM-1 as affected by time after excision (1, 4, 8 h) and temperature (20, 30, 40 °C) regime. ns: not significance, determined using a *t*-test (*p* > 0.05), and error bars represent standard deviations.

Independent-sample *t*-tests were employed to analyze pollen germination rates across the different treatment combinations. The results demonstrated that there were no statistically significant differences (*p* > 0.05) in in vitro pollen germination rates between the transgenic line SA6-8 and the non-transgenic control ZM-1 across all nine time–temperature treatment combinations (Figure 5) (Table A6). These findings indicate that the introduction of exogenous genes and the associated genetic transformation process did not exert significant adverse effects on the pollen germination capacity of the transgenic alfalfa line SA6-8, which performed consistently with the conventional material in terms of pollen viability. This study provides important experimental evidence for the environmental risk assessment of transgenic alfalfa, suggesting that the risk of pollen-mediated gene flow is not increased due to genetic modification.

## 3. Discussion

Successful in vitro pollen germination requires a carefully optimized culture medium, typically composed of carbohydrates, trace elements, and supplementary components such as sucrose, boric acid, calcium chloride, and potassium nitrate. In this study, we achieved an exceptionally high in vitro germination rate of over 90% for alfalfa pollen [31]. Notably, the medium formulation is simple and does not contain PEG4000, VB1, GA or other micronutrients [35,40,41,42]. Our experiments revealed that weather conditions and the initial selection of alfalfa florets significantly influence pollen germination rates. In contrast, sampling on cloudy days or from plants in poor physiological condition resulted in reduced reproducibility, with variation sometimes exceeding differences attributable to medium formulations.

In China, standard protocols exist for assessing in vitro pollen viability in crops like maize, cotton, and soybean [43]. These protocols primarily differ only in the culture medium composition, while key parameters such as the time after excision and incubation temperature are consistent. However, our study demonstrates significant interspecific variations in pollen viability and temperature adaptability. Consequently, these species-specific differences should be considered when formulating future standard protocols.

We believe this empirical approach can be extended to pollen viability studies in other plant species. Specifically, ensuring optimal environmental conditions and selecting healthy plants should be prioritized before conducting in vitro pollen viability assessments. We also conducted in vitro germination assays under outdoor conditions. Specifically, pollen was collected in Petri dishes and exposed to the outdoor environment for varying time intervals before germination tests were performed. The experiment was carried out at the High-Tech Agricultural Science Park of the Chinese Academy of Agricultural Sciences in Langfang, Hebei Province, China, starting at 2:00 PM. We observed that pollen completely lost viability after 16 h of exposure outdoors, culminating in a 0% germination rate. This result is substantially lower than that obtained under indoor controlled conditions, in which pollen remained viable even after 24 h post-excision. We hypothesize that this pronounced decline may be attributed to direct solar radiation (particularly UV exposure) or heavy dew deposition. These findings suggest that the viability of pollen from outcrossing species under natural conditions may be significantly lower than previously estimated from indoor experiments, where factors such as strong ultraviolet radiation, wind, and fluctuations in temperature and humidity are absent. This discrepancy raises an important question regarding the current environmental risk assessment framework for transgenic crops: whether the safety evaluation standards based on indoor data are overly stringent and may not accurately reflect realistic gene flow potential in field environments.

## 4. Materials and Methods

### 4.1. Plant Material and Pollen Collection

**Figure A1:**
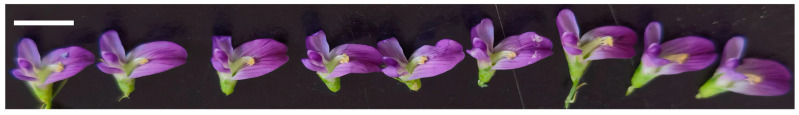
Selected alfalfa florets, bar = 5 mm.

**Figure A2:**
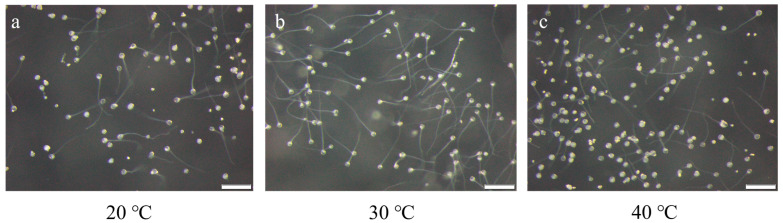
Pollen germination rate in response to different temperature. (**a**): In vitro germination of alfalfa pollen at 20 °C, bar = 200 μm. (**b**): In vitro germination of alfalfa pollen at 30 °C, bar = 200 μm. (**c**): In vitro germination of alfalfa pollen at 40 °C, bar = 200 μm.

In our preliminary experiments, we observed that alfalfa pollen exhibits relatively high viability, although initial pollen condition and weather factors significantly influence the germination rate. To ensure experimental consistency, a series of preliminary trials were conducted, leading to the establishment of a standardized sampling protocol: flowers of uniform size located in the upper part of the plant were collected between 10:00 a.m. and 4:00 p.m. on sunny days (Figure 1a,b and Figure A1). Specifically, flowers were selected based on the criterion that their anthers dehisced readily upon gentle touching of the keel petals with forceps (Figure 1c). The transgenic alfalfa line SA6-8 and its non-transgenic recipient ZM-1 were collected from the High-Tech Agricultural Science Park of the Chinese Academy of Agricultural Sciences, located in Langfang City, Hebei Province, China (latitude 39°32′ N, longitude 116°41′ E), and used as pollen donors. Conventional alfalfa seeds (ZM-1) and transgenic alfalfa seeds (SA6-8) were provided by the Biotechnology Research Institute of the Chinese Academy of Agricultural Sciences. Prior to the experiment, plants were cut at the base, and the stems were wrapped in moist cotton before being transported to the laboratory. Flowers from the upper part of the plants were selected for pollen germination assays. Experiments were conducted during July, when the monthly average temperature ranged from 22.1 °C to 31.6 °C. At the time of plant collection, the ambient temperature was 24–38 °C with a relative humidity of 60–75%. Pollen was subjected to in vitro germination assays immediately after collection.

### 4.2. Plant Material and Pollen Collection

All reagents used in the experiments were of analytical grade. The chemical reagents used in the culture medium preparation, including sucrose (CAS:57-50-1), H_3_BO_3_, (CAS:10043-35-3), CaCl_2_ (CAS:10043-52-4), KH_2_PO_4_ (CAS:7778-77-0), and MgSO_4_ (CAS:10034-99-8), were procured from Sinopharm Chemical Reagent Co., Ltd. (Shanghai, China).

### 4.3. Preparation and Optimization of Culture Media

All chemicals used were of analytical grade. Stock solutions were prepared by dissolving each component in deionized water at the following concentrations: sucrose 500 g/L, boric acid 2 g/L, calcium chloride 3 g/L, magnesium sulfate 1 g/L, and monopotassium phosphate 1 g/L. The stock solutions were filter-sterilized using a 0.22 μm PVDF membrane and stored at 4 °C for no longer than 30 days. Working solutions were prepared by diluting the stock solutions to the following final concentrations: sucrose (50, 75, 100, 125, 150, 200, and 400 g/L), boric acid (100, 200, and 300 mg/L), calcium chloride (30, 60, and 90 mg/L), monopotassium phosphate (0, 10, and 20 mg/L), and magnesium sulfate (0, 10, and 20 mg/L).

### 4.4. Temperature Treatment of Pollen

For the pollen germination assay, 10 mL of sterile liquid medium was pipetted into plastic plates measuring 35 mm in diameter. Alfalfa florets were inverted over the medium, and the keel petals were gently touched with fine forceps to release pollen grains into the medium. The dishes were then gently agitated to ensure uniform distribution of pollen across the surface. For each culture, 3–5 florets were collected, and pollen samples were assessed with three biological replicates. The plates were incubated in a precision temperature-controlled chamber (±1 °C) at graded temperatures: 15 °C, 20 °C, 25 °C, 30 °C, 35 °C, 40 °C, and 45 °C. After a 1 h incubation period, pollen germination was examined under a microscope.

### 4.5. Pollen In Vitro Treatment

For in vitro pollen processing, a dry filter paper was placed in a 35 mm Petri dish. Approximately 5–10 alfalfa florets (depending on pollen availability; minimal loss may occur during transfer) were evenly distributed over the dry filter paper. The dish was incubated at 30 °C in a constant-temperature incubator for a specified duration. After incubation, the filter paper was carefully removed, and the pollen was gently poured into a new 35 mm dish containing liquid culture medium. The dish was agitated gently to ensure uniform pollen distribution. Finally, the cultures were maintained stationary at 30 °C for 1 h.

### 4.6. Pollen Germination

All medium components in this study were dissolved in distilled water. To prevent component degradation caused by high-temperature sterilization, the medium was filter-sterilized using 0.22 μm membrane filters. A 10 mL aliquot of the medium was dispensed into 35 mm Petri dishes. Florets were inverted above the center of the medium, and the keel petals were gently touched with fine forceps to trigger the tripping mechanism—a characteristic feature of alfalfa flowers—thereby releasing pollen grains onto the medium surface. The dishes were then agitated gently to ensure even pollen distribution. The plates were incubated at 30 °C in a precision incubator for 1 h. After incubation, samples were observed under a Motic microscope. For each replicate, more than 20 pollen grains were imaged. Pollen was considered germinated when the tube length exceeded the grain diameter. Germination rates were quantified using ImageJ software (version 1.8.0).

### 4.7. Statistical Analysis

Three biological replicates were performed for each treatment group. Statistical analysis was performed using one-way ANOVA or t-tests in GraphPad Prism (version 8.0.2). The pollen germination rate was calculated using the following formula:

$$\mathrm{Pollen}\;\mathrm{germination}\;(\%)\;=\;\frac{Number\;of\;germinated\;pollen\;grains}{Total\;pollen\;grains\;counted}\;\times \;100$$

### 4.8. Microscopic Examination of Pollen Morphology

Pollen germination rates were observed using a Motic stereo microscope (Model moticam2506, Motic, Xiamen, China). The Petri dish was placed on the microscope stage, and the focusing knobs were adjusted to bring pollen grains into clear focus. Digital images were then acquired using the built-in camera system and saved in PNG format via Motic Images Plus 3.1 (×64) imaging software. For observation of the pollen germination process, incubated pollen samples were transferred to the concave area of a single-well slide using a micropipette, covered with a coverslip, and observed under 100× magnification.

## 5. Conclusions

This study establishes a standardized and efficient system for evaluating alfalfa pollen viability in vitro, addressing a critical methodological gap in the environmental risk assessment of transgenic alfalfa. The optimized culture conditions and a novel assessment protocol enabled a reliable comparative analysis, revealing no significant difference in pollen viability between the transgenic and non-transgenic lines under varied post-excision durations and temperatures.

The primary contribution of this work is the development of a practical and reproducible toolkit for quantifying pollen-mediated gene flow potential. This framework provides robust scientific data essential for the biosafety evaluation of transgenic alfalfa, thereby supporting more informed regulatory decision-making. Future research will focus on validating this protocol under field conditions and extending its application to other crop species.

## Figures and Tables

**Table 1:** Media used for the Sucrose concentration test.

Treatment	Sucrose (g/L)	H_3_BO_3_ (mg/L)	CaCl_2_ (mg/L)	Germination Rate (%)
M1	50	200	60	71.32 ± 2.31
M2	75	200	60	86.41 ± 2.49
M3	100	200	60	94.76 ± 3.27
M4	125	200	60	90.4 ± 1.74
M5	150	200	60	81.25 ± 1.37
M6	200	200	60	36.5 ± 6.07
M7	400	200	60	0 ± 0

**Table 2:** Media used for the Boric acid and calcium chloride concentration test.

Treatment	Sucrose (g/L)	H_3_BO_3_ (mg/L)	CaCl_2_ (mg/L)	Germination Rate (%)
M8	100	100	30	67.98 ± 4.01
M9	100	100	60	78.97 ± 4.45
M10	100	100	90	68.7 ± 4.22
M11	100	200	30	80.98 ± 2.42
M12	100	200	60	93.48 ± 1.62
M13	100	200	90	86.95 ± 1.29
M14	100	300	30	51.55 ± 3.13
M15	100	300	60	54.61 ± 2.06
M16	100	300	90	58.49 ± 1.44

**Table 3:** Media used for the Monopotassium phosphate and Magnesium sulfate concentration test.

Treatment	Sucrose (g/L)	H_3_BO_3_ (mg/L)	CaCl_2_ (mg/L)	KH_2_PO_4_ (mg/L)	MgSO_4_ (mg/L)	Germination Rate (%)
M17	175	200	60	0	0	91.62 ± 0.83
M18	175	200	60	10	10	98.24 ± 1.67
M19	175	200	60	10	20	94.17 ± 0.73
M20	175	200	60	20	10	94.2 ± 1.01
M21	175	200	60	20	20	92.77 ± 1.01

## Data Availability

All data generated or analyzed during this study are included in this published article.

## Abbreviations

The following abbreviations are used in this manuscript:

H_3_BO_3_	Boric acid
CaCl_2_	Directory of open access journals
KH_2_PO_4_	Monopotassium phosphate
MgSO_4_	Magnesium sulfate
GM	Genetically modified
GMOs	Genetically modified organisms
PNG	Portable network graphics

## Appendix A

**Table A1:** Pollen germination rate under different Sucrose concentration media.

Treatment	Germination Rate %		
M1	71.67	68.85	73.44
M2	88.33	83.61	87.30
M3	92.19	93.65	98.44
M4	90.00	88.89	92.31
M5	79.69	82.26	81.82
M6	43.33	34.43	31.75
M7	0.00	0.00	0.00

**Table A2:** Pollen germination rate under different Boric acid and calcium chloride concentration media.

Treatment	Germination Rate %		
M8	63.49	69.23	71.21
M9	73.85	81.82	81.25
M10	73.33	65.08	67.69
M11	78.33	81.54	83.08
M12	93.33	91.94	95.16
M13	85.48	87.50	87.88
M14	48.44	51.52	54.69
M15	56.45	55.00	52.38
M16	58.33	57.14	60.00

**Table A3:** Pollen germination rate under different Monopotassium phosphate and Magnesium sulfate concentration media.

Treatment	Germination Rate %		
M17	91.67	90.77	92.42
M18	96.67	98.06	100.00
M19	95.00	93.65	93.85
M20	93.33	95.31	93.94
M21	92.19	92.19	93.94

**Table A4:** Pollen germination rate under different temperature.

Temperature (°C)	Germination Rate %		
15	0.00	0.00	0.00
20	37.10	29.23	37.31
25	85.07	92.42	95.38
30	95.08	98.51	99.12
35	97.01	90.63	95.45
40	49.23	58.46	55.22
45	1.67	6.06	11.94

**Table A5:** Pollen germination rates following varying post-excision durations.

Post-Excision Time (min)	Germination Rate %		
0	98.44	96.77	97.01
1	98.51	98.48	96.88
2	95.52	98.39	96.97
4	88.71	92.06	93.94
6	79.03	78.46	83.33
8	64.52	68.25	61.19
12	44.44	57.58	41.79
24	17.19	19.40	15.63

**Table A6:** Pollen viability of transgenic alfalfa SA6-8 versus non-transgenic ZM-1 under varying post-excision durations and temperature regimes.

Plant Material	Temperature (°C)	Post-Excision Time (min)	Germination Rate %		
SA6-8	20	1	40.30	33.85	36.51
ZM-1	20	1	34.92	41.94	30.65
SA6-8	20	4	25.00	32.84	34.33
ZM-1	20	4	36.36	32.81	30.77
SA6-8	20	8	21.88	26.98	27.27
ZM-1	20	8	22.39	25.81	26.56
SA6-8	30	1	96.77	95.31	98.39
ZM-1	30	1	95.52	96.97	100.00
SA6-8	30	4	88.71	94.03	92.06
ZM-1	30	4	90.32	89.23	87.10
SA6-8	30	8	62.69	66.67	60.61
ZM-1	30	8	60.66	63.93	56.72
SA6-8	40	1	36.36	46.97	41.79
ZM-1	40	1	45.45	37.31	47.69
SA6-8	40	4	5.97	16.42	19.70
ZM-1	40	4	20.00	15.63	12.50
SA6-8	40	8	3.08	4.48	0.00
ZM-1	40	8	1.59	3.33	3.17
